# Gender-based violence and its health risks on women in Yaoundé, Cameroon

**DOI:** 10.1186/s13690-024-01308-2

**Published:** 2024-06-18

**Authors:** Georges Nguefack-Tsague, Adidja Amani, Valérie Djouna Dadjie, Donato Koyalta, Debora Nounkeu Carole, Fanny Nadia Dissak-Delon, Fabrice Zobel Lekeumo Cheuyem, Gilles Protais Lekelem Dongmo, Christelle Mbe Anastasie, Justine Laure Menguene Mviena, Odette Kibu, Marie Nicole Ngoufack, Magloire Biwole Sida, Catherine Juillard, Alain Chichom-Mefire

**Affiliations:** 1 Department of Public Health, Faculty of Medicine and Biomedical Sciences, University of Yaoundé I, Yaoundé, Cameroon; 2 Higher Institute of Medical Technology, Yaoundé, Cameroon; 3 Département de la Microbiologie, Faculté des sciences de la santé humaine, Université de Ndjamena, Ndjamena, Tchad; 4 Ministry of Public Health, Yaoundé, Cameroon; 5 Department of Public Health, Faculty of Health Sciences, University of Bamenda, Bamenda, Cameroon; 6 Challenges Initiative Solutions, Yaoundé, Cameroon; 7 University of Hasselt, Diepenbeek, Belgium; 8 Department of Public Health and Hygiene, Faculty of Health Sciences, University of Buea, Buea, Cameroon; 9 Department of Surgery, Program for the Advancement of Surgical Equity, University of California, Los Angeles, United States of America; 10 Department of Surgery, Faculty of Health Sciences, University of Buea, Buea, Cameroon

**Keywords:** Gender-based violence, Health effects, Women’s health, Adverse health outcome, Types of violence

## Abstract

**Introduction:**

Gender-based violence (GBV) is a major public health problem that disproportionately affects women. In Cameroon, as well as other countries worldwide, GBV has immediate effects on women’s health, with one in three women experiencing physical or sexual violence from an intimate partner, affecting their physical and reproductive health. The objective of this study was to determine the health risks associated with GBV among women in Yaoundé.

**Methods:**

A cross-sectional study was conducted in Yaoundé (Cameroon), from August to October 2022. Adverse health outcome included mental disorders, physical trauma, gynaecological trauma, behavioral disorders, and any other disorder. Tests of associations were used to establish relationships between qualitative variables. Associations were further quantified using crude odds ratio (OR) for univariate analysis and adjusted odds ratio (aOR) for multivariate analysis with 95% confidence interval (CI). Independent variables included: Physical violence, Sexual violence, Economic violence, Emotional violence, Age, Number of children, and Marital status. Variables with *p*-value˂0.05 were considered statistically significant.

**Results:**

A total of 404 women aged 17 to 67 years were interviewed. Emotional violence was the most commonly reported violence (78.8%), followed by economic violence (56.9%), physical violence (45.8%) and sexual violence (33.7%). The main reasons for violence were jealousy (25.7%), insolence (19.3%) and the refusal to have sexual intercourse (16.3%). The prevalences of adverse health outcomes were physical trauma (90.9%), followed by mental disorders (70,5%), gynaecological trauma (38.4%), behavioral disorders (29.7%), and other (5.5%). Most victims reported at least one of the above-mentioned conditions (80.2%). Women who were victims of any kind of violence had a higher likelihood of experiencing adverse health outcomes: physical violence [OR = 34.9, CI(10.8-112.9), *p* < 0.001]; sexual violence [OR = 1.5, CI(0.9–2.7), *p* = 0.11]; economic violence [OR = 2.4, CI(1.4–3.9), *p* = 0.001]; and emotional violence [OR = 2.9, CI(1.7–4.9), *p* < 0.001]. Using multiple binary logistic regression, only physical violence [aOR = 15.4, CI(6.7–22.5), *p* = 0.001] remained highly associated with an increased likelihood of having adverse health outcomes.

**Conclusion:**

This study underscores the urgent need for comprehensive interventions to address GBV, including improved reporting and documentation of cases, increased awareness among healthcare providers, the establishment of support networks for victims, primary and secondary prevention of GBV. It is essential that the Government of Cameroon, through the Ministries in charge of Health and Women’s Empowerment, minimizes the health effects of GBV through early identification, monitoring, and treatment of GBV survivors by providing them with high-quality health care services.

## Background

Gender-based violence (GBV) is a global public health and social problem that affects women, girls, men, and boys and takes different forms such as physical, sexual, psychological, emotional, and social violence [1]. GBV is defined as a violence that is directed at an individual based on his or her biological sex, gender identity, or his or her perceived adherence to socially defined norms of masculinity and femininity [2]. GBV takes place in a context of systemic domination, which explains why most violence against women is perpetrated by men. The global prevalence of GBV remained between 30% and 35% from 2005 to 2019 [3]. Almost one in three women worldwide have experienced physical or sexual violence inflicted by someone other than a partner, or both [4]. GBV results in minor or severe physical, psychological, and social harm than can have direct impacts on women’s physical and mental health, such as bodily injuries, as well as indirect impacts, such as chronic health problems resulting from prolonged stress [5]. Thus, having been a victim of violence is a risk factor for various ailments and diseases such as headaches, anxiety, depression, sexually transmitted diseases, HIV, and stroke [6–8]. The more severe the violence, the greater the impact on a woman’s physical and mental health, and the effects of different types and multiple episodes of violence appear to be cumulative over time [9, 10]. GBV is a pervasive public health and social problem that affects the quality of life of victims [11]. The literature reveals that the problem is most pronounced in developing countries with low-socioeconomic status and limited education, especially in sub-Saharan Africa (SSA) [12–16].

Regarding the institutional framework for the fight against GBV in Cameroon; in accordance with Decree N°2011/408 of December 09, 2011 on the organization of the Government, the Ministry of Women’s Empowerment and the Family is responsible for the development and implementation of measures relating to the respect of women’s rights and the protection of the family. Decree N°2012/638 of December 21, 2012, creates within this Ministry a Department for the Promotion and Protection of the Family and Children’s Rights, with certain responsibilities relating to the prevention and management of conjugal, domestic and family violence. Decree N°2005/160 of May 25, 2005 on the organization of the Ministry of Social Affairs assigns to it; among other missions; the social protection of children, the elderly and the disabled. The Ministry of Justice, whose courts enforce laws against conjugal, domestic and family violence. The Ministry of Public Health, whose health units provide care for survivors of violence. Among other actors, the National Commission on Human Rights and Freedoms, which can be contacted by any citizen in the event of human rights violations; the Secretary of State for Defense, whose services conduct judicial investigations in cases of violence against citizens, and are responsible for the physical protection of survivors; the General Delegation for National Security, which handles complaints relating to conjugal, domestic and family violence, including the physical protection of individuals. In addition, several development partners support the government’s efforts to combat conjugal, domestic and family violence. The government’s efforts are usefully complemented by a host of civil society organizations working in general to protect human rights, and in particular those of the most vulnerable.

In addition, Cameroon has a national strategy to combat gender-based violence, based on the combined action of all those involved. Encouraging results have been achieved; for example, in the context of prevention, actions have focused on behavior change communication; mobilization and structuring of local communities and village committees; advocacy and lobbying; and training, including for law enforcement officers. Previous studies on GBV and its impact on health have focused on the prevalence of sexual violence among sex workers and refugees, its association with HIV risk, antiretroviral treatment interruption and women’s reproductive health [17–21]. Despite the country’s commitment to Sustainable Development Goal N^o^3 on good health and well-being and Goal N^o^5 on gender equality, women are still more vulnerable to violence [22]. However, studies on the health effects of GBV are scarce in Cameroon, especially in Yaoundé, the capital of Cameroon which carries the heaviest burden at 64% according to the Cameroon Demographic and Health Survey 2018 [23]. To the best of our knowledge, no study has examined the health effects of GBV in Yaoundé. Therefore, the present study aimed to fill this gap by identifying the health risks associated with GBV in Yaoundé.

## Methodology

### Study design and setting

This was a cross-sectional study, conducted in Yaoundé, the capital of Cameroon from August to October 2022. According to the World Bank Classification, Cameroon is a low-middle-income country in Central Africa with a Gender Inequality Index rank of 141 [24]. Yaoundé is the second most populous city in Cameroon, with a population of 4.5 million [25].

### Study population and eligibility criteria

The study population consisted of all victims registered in two organizations that implement GBV interventions in Yaoundé and who gave their informed consent to participate.

### Sampling

All eligible women (428) of these two organizations were contacted for the study; about 95% (404) of victims of GBV consented to participate in the study.

### Variables

The main variables included age, level of education, region of origin, marital status, religion, professional situation, types of gender-based violence (emotional violence, physical violence, sexual violence, and economic violence). Health effect included mental disorders, physical trauma, gynaecological trauma, behavioral disorders, and any other disorder. It was code as 1 (yes) if an individual had at least one of the above-mentioned conditions.

### Operational definitions

Physical violence was defined as any acts like slapping, or throwing something that could hurt; pushing or shoving; hitting with a fist or something else that could hurt; kicking, dragging or beating; choking or burning; threating with a gun, knife or other weapon [26]. Emotional violence was defined as being insulted or made to feel bad about oneself; being humiliated or belittled in front of others; being intimidated or scared on purpose (for example by a partner yelling and smashing things); being threatened with harm (directly or indirectly in the form of a threat to hurt someone the respondent cared about) [26]. Sexual violence was defined as being physically forced to have sexual intercourse against her will; having sexual intercourse because she was afraid of what her partner might do; being forced to do something sexual she found degrading or humiliating [26]. Economic violence was defined as the acts of denying her intimate partner access to financial resources, typically as a form of abuse or control or in order to isolate her or to impose other adverse consequences to her well-being [27]. Mental disorder is characterized by a clinically significant disturbance in an individual’s cognition, emotional regulation, or behaviour; as described by the International Classification of Diseases 11th Revision (ICD-11). Gynecologic trauma was referred as any injury to the female genital area while Physical trauma was referred as any serious injury to the body. Behavioral disorder was defined as a disability characterized by behavioral or emotional responses that are not accepted by the norm and referent group and have adverse effects on educational performance; and can affect both children and adults [28].

### Data processing and analysis

Data were collected and recorded using an Epi-collect 5 mobile application, and were analyzed using IBM-SPSS (version 26.0). Categorical variables were presented as numbers and percentages, and quantitative variables expressed as mean and standard deviation (since they all do not deviate from normal distribution). In bivariate analysis, the associations between variables were assessed by the Chi-square test (or Fisher’s exact test where applicable) and quantified using Odds Ratios (OR) with 95% confidence interval (CI). To account for confounding variables, associations were assessed using a multiple binary logistic regression model and quantified using adjusted odds ratios (aOR). Independent variables included: Physical violence, Sexual violence, Economic violence, Emotional violence, Age, Number of children, and Marital status. Variables with *p*-values less than 0.05 were considered statistically significant.

### Ethical considerations

The researchers addressed a request for research authorization to the chairpersons of the two organizations implementing GBV interventions in Yaoundé. After obtaining the necessary authorizations, the researchers were granted permission to access the registers and contact details of the victims. During the interviews, the researchers explained the purpose of the study to the victims and obtained their informed consent to participate followed by the collection of data. The study was approved by the Centre Regional Ethics Committee for Research in Human Health (Ethical approval number: CE-N°00709/CRERSHC/2022; July 26, 2022) and the University of Douala (Ethical approval number: 3307/CEI _Udo/06/22T; June 22, 2022). The study was conducted in accordance with the ethical principles of the World Medical Association’s Declaration of Helsinki. Participants personal identities and other private information were anonymized.

## Results

### Socio-demographic profile of victims

Table 1 presents the distribution of 404 victims of GBV according to their socio-demographic characteristics. Participants aged 17–67 years with an average of 30.8 ± 8.3 years, with age group 20–29 years being the most represented (46.9%). Most of victims had completed secondary education (57.9%), 13.4% had primary education (13.4%) and 6.9% had no formal education (6.9%). Most of victims were single (60.4%) and 17.1% were married in monogamous relationships. Half (52.2%) of victims of GBV were unemployed. Almost half of them were catholic (49.3%). Four over ten (41.1%) were from the Centre region (41.1%), followed by the West (14.4%), and Littoral (11.4%). About 45% of the victims reported a history of family violence. Regarding fear of their husband/partner, 57.7% reported being sometimes afraid while 21.3% reported never being afraid.

**Table 1:** Socio-demographic characteristics of GBV victims in Yaoundé

Variables	Frequency (*n*)	Percentage (%)
**Age (years)**		
17–19	15	3.7
20–24	91	22.5
25–29	99	24.5
30–34	86	21.3
35–39	52	12.9
40–67	61	15.1
**Education**		
None	28	6.9
Primary	54	13.4
Secondary	234	57.9
More than secondary	88	21.8
**Marital status**		
Single	244	60.4
Married (Monogamy)	69	17.1
Married (Polygamy)	16	4.0
Divorced/separated	56	13.8
Widow	19	4.7
**Profession**		
Unemployed	211	52.2
Employed	188	46.5
Retired	5	1.3
**Religion**		
Catholic	199	49.3
Protestant	91	22.5
Other Christians (Pentecostal, Jehovah’s Witness, Adventist Revival Church)	66	16.3
Muslim	33	8.2
Any	14	3.5
Animist	1	0.2
**Region**		
Centre	166	41.1
West	58	14.4
Littoral	46	11.4
East	35	8.7
North	34	8.4
Far North	25	6.2
South West	16	4.0
South	8	2.0
Adamawa	8	2.0
North West	7	1.8

### Prevalence of violence and health effects

The most prevalent type of violence was emotional (78%), followed by economic violence (56.9%) and physical violence (45.8%). One-third of victims reported sexual violence (33.7%). Social violence was the least commonly reported type of violence (8.2%).

**Fig. 1:**
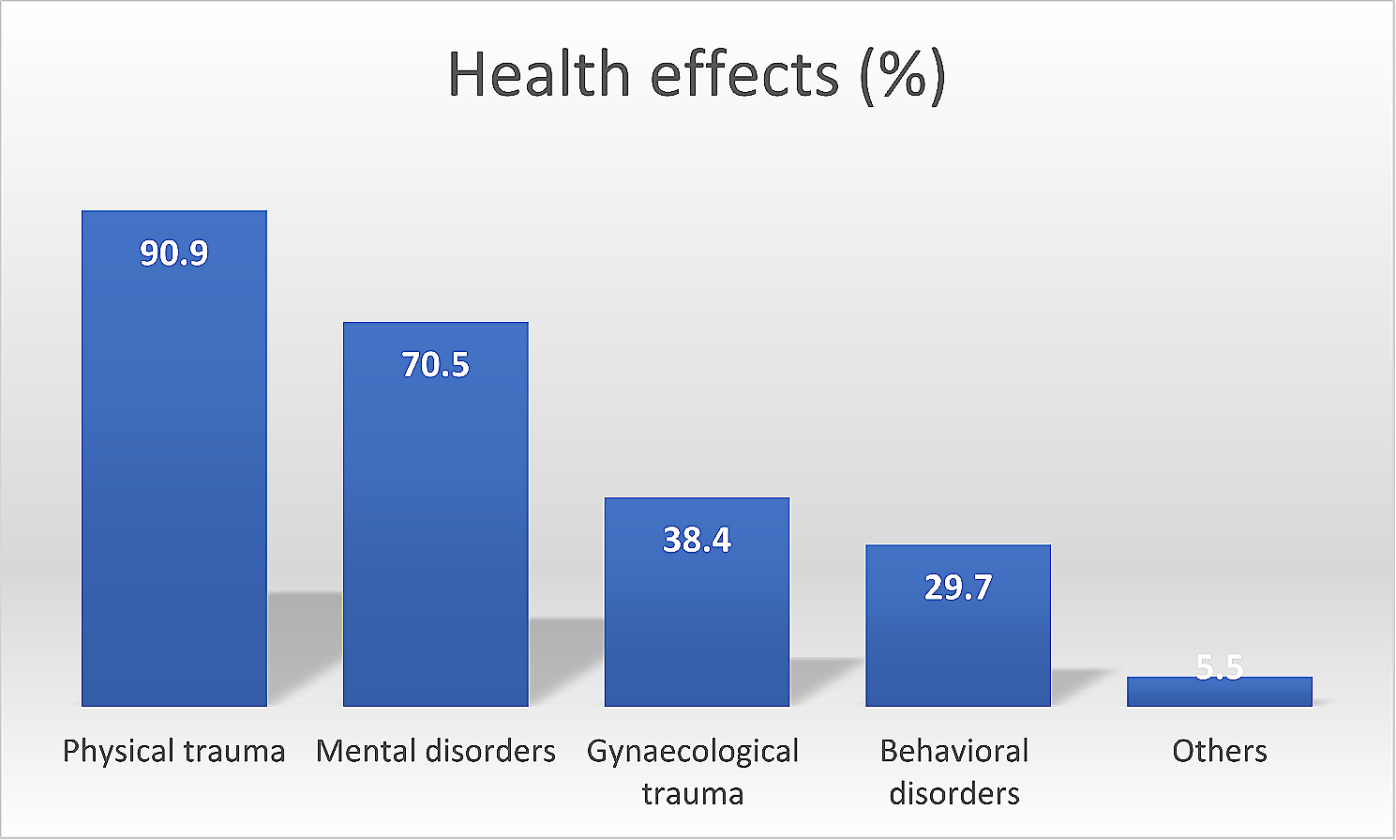
Health effects of GBV

Figure 1 shows that the most prevalent health effect was physical trauma (90.9%), followed by mental disorders (70,5%), gynaecological trauma (38.4%), and behavioral disorders (29.7%).

Among the victims, 80.2% reported experiencing at least one health effect, while 19.8% reported no health effects.

### Reasons for GBV

The main reasons for violence (Table 2), according to the victims, were jealousy (25.7%), insolence (19.3%), and refusal to have sex (16.3%).

**Table 2:** Main reasons for violence according to the victims

Main reasons according to the victim of the violence	*n*	Percentage (%)
Jealousy	104	25.7
Insolence	78	19.3
Refusal to have sex	66	16.3
Refusal of advances	34	8.4
Sexual dissatisfaction	31	7.7
Refusal of certain sexual practices	18	4.5
Contempt	12	3.0
Refusal to share the wife’s property	6	1.5
Don’t know	49	12.1
Other (s) to be specified*	6	1.5

*refusal to share custody of the child, refusal to pay, refusal to stop working, refusal of money, and lack of respect for his mother

### Association between health effects and socio-demographic characteristics

The likelihood of having health effects increased significantly with age (*p* < 0.001). In particular, age group 40–67 years had higher proportions (88.5%) of victims experiencing health effects compared to 17–19 years [OR = 6.8, CI(1.9–24.4), *p* = 0.004], and were more likely to have health effects. Among the marital status categories, victims who were divorced/separated (83.9%) and those in polygamous marriages (93.8%) had higher proportions of health effects (Table 3).

**Table 3:** Association between health effects and socio-demographic characteristics

Variables	Health effects (*n*(%))			OR (CI)	*p*-value
	At least one health effect 324 (80.2)	No health effect 80 (19.8)	Total *N* = 404		
**Age (years)**					
17–19	8 (53.3)	7 (46.7)	15(100.0)	1	1
20–24	66 (72.5)	25 (27.5)	91(100.0)	2.3(0.8-7.0)	0.141
25–29	78 (78.8)	21 (21.2)	99(100.0)	3.3 (1.1–10.0)	0.040
30–34	75 (87.2)	11 (12.8)	86(100.0)	6.0 (1.8–19.7)	0.003
35–39	43 (82.7)	9 (17.3)	52(100.0)	4.2 (1.2–14.5)	0.024
40–67	54 (88.5)	7 (11.5)	61(100.0)	6.8 (1.9–24.4)	0.004
**Education**					
None	25 (89.3)	3 (10.7)	28 (100.0)	1	1
Primary	47 (87.0)	7 (13.0)	54 (100.0)	0.8(0.2–3.4)	0.768
Secondary	177 (75.6)	57 (24.4)	234 (100.0)	0.4 (0.1–1.3)	0.117
More than secondary	75 (85.2)	13 (14.8)	88 (100.0)	0.7(0.2–2.6)	0.589
**Marital status**					
Single	187 (76.6)	57 (23.4)	244 (100.0)	1	1
Divorced/separated	47 (83.9)	9 (16.1)	56 (100.0)	1.6(0.7–3.4)	0.238
Monogamous	60 (87.0)	9 (13.0)	69 (100.0)	2.0(1.0-4.3)	0.068
Polygamous	15 (93.8)	1 (6.3)	16 (100.0)	4.6(0.6–35.4)	0.145
Widow	15 (78.9)	4 (21.1)	19 (100.0)	1.1(0.4–3.6)	0.819
**Number of children**					
0	48 (65.8)	25 (34.2)	73(100.0)	0.3(0.1–0.9)	0.030
1–2	172 (81.5)	39 (18.5)	211(100.0)	0.7(0.3–1.9)	0.507
3–4	73 (86.9)	11 (13.1)	84(100.0)	1.1(0.3–3.3)	0.907
≥ 5	31 (86.1)	5 (13.9)	36(100.0)	1	1
**Profession**					
Unemployed	164 (77.7)	47 (22.3)	211(100.0)	0.7(0.5–1.2)	0.241
Retirement	5 (100.0)	0 (00.0)	5 (100.0)	NA	NA
Employed	155 (82.4)	33 (17.6)	188(100.0)	1	1

### Association between types of violence and health effects

The likelihood of experiencing health effects was significantly higher among women who experienced physical violence [OR = 34.9, CI(10.8-112.9), *p* < 0.001], economic violence [OR = 2.4, CI(1.4–3.9), *p* = 0.001], and emotional violence [OR = 2.9, CI(1.7–4.9), *p* < 0.001] (Table 4).

**Table 4:** Association between health effects and types of violence

Types of violence	Health effects (*n*(%))			OR(CI)	*P*-Value
	At least one health effect 324 (80.2)	No health effect 80 (19.8)	Total = 404		
**Physical violence**					
No	139 (63.5)	80 (36.5)	219 (100.0)	1	
Yes	182 (98.4)	3 (1.6)	185 (100.0)	34.9(10.8-112.9)	**< 0.001**
**Sexual violence**					
No	209 (78.0)	59 (22.0)	268(100.0)	1	
Yes	115 (84.6)	21 (15.4)	136(100.0)	1.5 (0.9–2.7)	0.111
**Economic violence**					
No	126 (72.4)	48 (27.6)	174(100.0)	1	
Yes	198 (86.1)	32 (13.9)	230(100.0)	2.4 (1.4–3.9)	**0.001**
**Emotional violence**					
No	58 (65.2)	31 (34.8)	89(100.0)	1	
Yes	266 (84.4)	49 (15.6)	315(100.0)	2.9(1.7–4.9)	**< 0.001**

### Multivariate analysis of the association between health effects and types of violence

Table 5 presents the results of the multiple binary logistic regression of the association between health effects and significant variables at univariate level. Physical violence remained significantly associated with an increased likelihood of experiencing health effects (aOR = 15.4, CI(6.7–22.5), *p* = 0.001).

**Table 5:** Association between health effects and types of violence (Multivariate analysis)

Variables	OR (95% CI)	aOR(95% CI)	*P*-Value	
Physical violence	34.9(10.8–112.9)	15.4(6.7–22.5)	**0.001**	
Sexual violence	1.5(0.9–2.7)	1.1(0.5–2.1)	0.831	
Economic violence	2.4(1.4–3.9)	1.3(0.7–2.6)	0.399	
Emotional violence	2.9(1.7–4.9)	1.1(0.6–2.2)	0.763	
40–67 years	6.8(1.9–24.4)	9.9(1.5–67.1)	**0.019**	
No child	0.3(0.1–0.9)	0.7(1.2–3.5)	0.709	
Monogamy	2.0(1.0-4.3)	1.2(0.5–3.2)	0.657	

## Discussion

This present study aimed to fill this gap by identifying the health risks associated with GBV in Yaoundé. A significant proportion of victims had completed secondary education. This underscores the need for targeted interventions and support for these specific groups. This finding is similar to several studies conducted in sub–Saharan Africa reporting varying prevalence rates of sexual and physical violence among women with the same age group and level of education, ranging from 15 to 71% [4, 14, 29–31]. The predominance of single victims suggests that GBV can occur within various relationship contexts, emphasizing the importance of addressing GBV beyond marital relationships. These findings align with previous researchers indicating the widespread nature of GBV and its impact on individuals across different demographic backgrounds [32, 33]. In fact, single women certainly have sexual partners who probably disrespect and despise them leading to GBV. Men give less consideration to women who are not their wives when they are involved in an intimate relationship.

The study also found that most of victims were unemployed, highlighting the impact of GBV across different socio-cultural backgrounds [34–36]. Unemployed women or those who have lost their jobs cannot support their partners in providing for the family’s economic needs. This uncomfortable situation can make men to become violent. The prevalence of domestic violence can have a significant impact on economic activity, with a 1% increase in violence against women associated with a 9% decrease in economic activity [37]. Regarding the reasons for GBV, jealousy emerged as the most prevalent reason, followed by insolence and refusal to have sex. The high prevalence of jealousy as a reason for GBV aligns also with previous research on the topic [38]. Jealousy can stem from feelings of insecurity, possessiveness, and a desire to control one’s partner. The prevalence of fear among victims towards their husband/partner underscores the psychological impact of GBV.

The association between health effects and socio-demographic characteristics of GBV victims revealed that victims who were older, had more children, and had no formal education were more likely to experience health effects. This may be due to the fact that older victims may have been exposed to GBV for a longer period of time, while victims with more children may have more responsibilities and less time to take care of their health [39–42].

This study suggests that women who experience physical, economic violence, and emotional violence are more likely to experience health problems. The high odds ratio for physical violence suggests that women who experience physical violence are at a significantly higher risk of experiencing health problems. Our findings corroborates those of previous studies which have shown that women who experience violence are more likely to experience adverse health outcomes [43]. Physical violence resulted in injury in 41.5% of female victims which is consistent with previous reports highlighting that physical violence is associated with a range of negative health outcomes, including injuries, chronic pain, and mental health problems [44–47]. In addition, GBV led to sexual and reproductive health effects, including unwanted pregnancies, induced abortions, gynecological problems, and sexually transmitted infections, including HIV [48].

Violence is very common in society, but most healthcare providers fail to diagnose and record it due to socio-cultural and traditional barriers, lack of time, resources, inadequate physical facilities, lack of awareness, knowledge, and poor clinical practice with limited direct communication and inability to perform a complete physical examination, let alone record and monitor the effectiveness and quality of care. In addition, fear of violence and stigma reduce the willingness of many victims to seek health care. The vast majority turn to informal networks of friends and community members for help.

Overall, the findings of this study underscore the need to consider socio-demographic characteristics when developing interventions to prevent and respond to GBV. Comprehensive interventions that address the multiple forms of violence that women may experience should involve a range of stakeholders, including health care providers, social workers, law enforcement officials, and community members, and should prioritize the safety and well-being of survivors of GBV.

### Strengths and limitations

Strengths of this study include its comprehensive analysis of the relationship between different types of violence and health effects, and its focus on a specific geographical region. The study provides valuable insights into the prevalence and impact of GBV on women’s health in Yaoundé. However, there are a number of limitations to consider. The study’s cross-sectional design limits the ability to establish causality between GBV and health effects. Findings relied on self-reported data, which may be subject to recall bias, social desirability bias and underreporting due to the sensitive nature of the topic. In addition, the study only included women in Yaoundé, Cameroon, and therefore, the findings may not be generalizable to other populations. Despite these limitations, the study provides important insights into the association between different types of violence and health outcomes among women. Future research could benefit from longitudinal designs and measures of health outcomes to further explore the complex relationship between GBV and women’s health.

## Conclusions

This research paper found that older adults, single, unemployed women were most vulnerable to experience adverse health outcomes following GBV. Emotional, economic, physical, and sexual violence were the most common forms of GBV. These findings underscore the urgent need to address GBV as a major public health concern. The Government of Cameroon, through the Ministries of Public Health, Women’s Empowerment and the Family, and Social Affairs, must take action to provide quality health services to survivors through early identification, monitoring, and treatment. Efforts should focus on raising awareness, improving access to healthcare services, and implementing policies and programmes to prevent and respond to GBV. By addressing the underlying factors contributing to GBV and providing support to victims, it is possible to mitigate the adverse health effects and promote the well-being of women in the community.

## Data Availability

No datasets were generated or analysed during the current study.

## Abbreviations

aOR: adjusted odds ratio
GBV: Gender-Based Violence
HIV: Human Immunodeficiency Virus
IPV: Intimate Partner Violence
OR: odds ratio
CI: Confidence interval
SSA: sub-Saharan Africa
WHO: World Health Organization
